# Cranio-Vertebral Junction Triangular Area: Quantification of Brain Stem Compression by Magnetic Resonance Images

**DOI:** 10.3390/brainsci11010064

**Published:** 2021-01-06

**Authors:** Chih-Chang Chang, Ching-Lan Wu, Tsung-Hsi Tu, Jau-Ching Wu, Hsuan-Kan Chang, Peng-Yuan Chang, Li-Yu Fay, Wen-Cheng Huang, Henrich Cheng

**Affiliations:** 1 Department of Neurosurgery, Neurological Institute, Taipei Veterans General Hospital, Taipei 11217, Taiwan; ccchang74@gmail.com (C.-C.C.); jauching@gmail.com (J.-C.W.); hsuankanchang@gmail.com (H.-K.C.); leofay1978@gmail.com (L.-Y.F.); wchuang518@gmail.com (W.-C.H.); henrichch@gmail.com (H.C.); 2 School of Medicine, National Yang-Ming University, Taipei 11217, Taiwan; clwu423@gmail.com (C.-L.W.); acidbummer@gmail.com (P.-Y.C.); 3 Department of Biomedical Engineering, National Yang-Ming University, Taipei 11217, Taiwan; 4 Department of Radiology, Taipei Veterans General Hospital, Taipei 11217, Taiwan; 5 Taoyuan General Hospital, Ministry of Health and Welfare, Taoyuan 32748, Taiwan

**Keywords:** cervical triangular area, odontoid fracture, rheumatoid arthritis (RA), atlantoaxial subluxation (AAS), basilar invagination (BI), os odontoideum, craniovertebral junction (CVJ)

## Abstract

(1) Background: Most of the currently used radiological criteria for craniovertebral junction (CVJ) were developed prior to the popularity of magnetic resonance images (MRIs). This study aimed to evaluate the efficacy of a novel triangular area (TA) calculated on MRIs for pathologies at the CVJ. (2) Methods: A total of 702 consecutive patients were enrolled, grouped into three: (a) Those with pathologies at the CVJ (*n* = 129); (b) those with underlying rheumatoid arthritis (RA) but no CVJ abnormalities (*n* = 279); and (3) normal (control; *n* = 294). TA was defined on T2-weighted MRIs by three points: The lowest point of the clivus, the posterior-inferior point of C2, and the most dorsal indentation point at the ventral brain stem. Receiver operating characteristic (ROC) analysis was used to correlate the prognostic value of the TA with myelopathy. Pre- and post-operative TA values were compared for validation. (c) Results: The CVJ-pathology group had the largest mean TA (1.58 ± 0.47 cm^2^), compared to the RA and control groups (0.96 ± 0.31 and 1.05 ± 0.26, respectively). The ROC analysis calculated the cutoff-point for myelopathy as 1.36 cm^2^ with the area under the curve at 0.93. Of the 81 surgical patients, the TA was reduced (1.21 ± 0.37 cm^2^) at two-years post-operation compared to that at pre-operation (1.67 ± 0.51 cm^2^). Moreover, intra-operative complete reduction of the abnormalities could further decrease the TA to 1.03 ± 0.39 cm^2^. (4) Conclusions: The TA, a valid measurement to quantify compression at the CVJ and evaluate the efficacy of surgery, averaged 1.05 cm^2^ in normal patients, and 1.36 cm^2^ could be a cutoff-point for myelopathy and of clinical significance.

## 1. Introduction

The craniovertebral junction (CVJ) has a unique anatomy, which is composed of bony structural support and ligamentous connections for motility, and is responsible for the majority of head movements in humans. Due to the highly mobile nature and the featured condylar joints with flat and round articular surfaces, the CVJ is subject to degeneration, instability, and vulnerable to trauma [1,2]. The CVJ is also essential in structural support of the skull and for bony protection to neuronal tissues, including the brain stem and spinal cord. Thus, deformity or instability of the CVJ is frequently comorbid with spinal cord or brain stem injury. The common non-neoplastic pathologies of the CVJ include odontoid fracture, os odontoideum, basilar invagination (BI), atlantoaxial subluxation (AAS), and pannus formation caused by rheumatoid arthritis (RA). RA is the most common inflammatory disease of the cervical spine, with the CVJ as the most commonly involved location. The complex joints of the CVJ are susceptible to the arthropathy of RA, which leads to the destruction of ligaments, joints, and bones, typically presenting in the form of AAS and BI. The surgical indication and timing remain a challenging topic for the management of RA-related CVJ pathologies. There have been numerous radiological diagnostic criteria for disorders of the CVJ for many decades, even earlier than the invention of magnetic resonance imaging (MRI), including atlantodental interval (ADI), posterior atlantodental interval (PADI) for AAS [3,4], Chamberlain line, McRae line, and McGregor line for BI [5]. All of these radiological measurements are based on bony landmarks identified on plain radiographs, and unanimously require Roentgenology exposures. Although the fluoroscopic examinations allow rapid and real-time evaluation, some of the radiographic landmarks are better appreciated on computed tomography (CT) scans and might be ambiguous on plain radiographs. Furthermore, these radiological parameters are indirect measurements of the brain stem or spinal cord, because the image quality of neural and soft tissues are suboptimal. In recent decades, the extent of compression to the medulla and spinal cord has been easily demonstrated by MRI, which has become the standard of care. Due to the complexity of anatomy in the CVJ, MRI is commonly adapted for such disorders, including trauma, congenital anomaly, malalignment, inflammation, and neoplasms.

**Figure 1:**
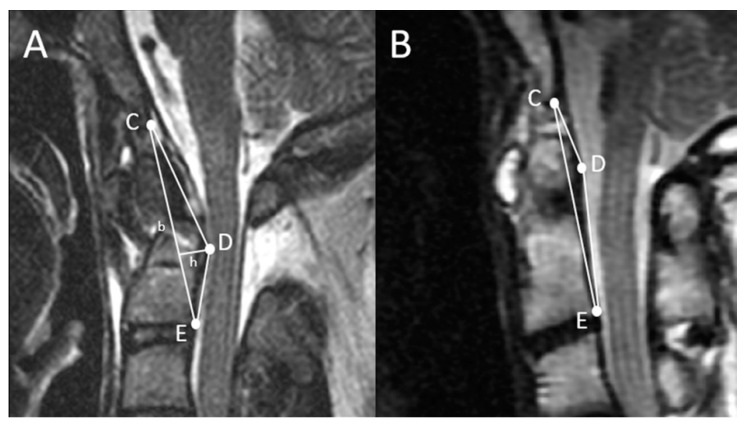
Proposed assessment of ventral compression at the craniovertebral junction (CVJ) by using a simulated triangle. The three points of the triangle are the lowest point of the clivus (C), the posterior-inferior point of the axial vertebral body (E), and the most indenting point of the pathology (D). The triangular area can be calculated as follows: (b × h)/2. Demonstration of the pre-operation (**A**) and post-operation (**B**) triangular area (TA) after atlantoaxial fixation.

MRI is the diagnostic modality of choice for disorders at the CVJ because MRI overcomes the limitation of X-rays in soft tissue differentiation. For example, in patients with RA who present with retro-dental pannus formation, the spinal cord compression can hardly be appreciated by radiographs, but are easily depicted by MRI. Moreover, the conventional X-ray based radiological measurements of CVJ anomalies are infrequently correlated with the prognosis of management, since soft tissues and nerves are hardly taken into account. To mitigate the above-mentioned shortcomings, the current study aimed to validate an MRI-based measurement used for the quantification of neural tissue compression at the CVJ and evaluation of the efficacy of its surgical management. The triangular area (TA) of the CVJ, proposed by Chang et al. [6], is a novel measurement that can quantify the degree of compression caused by basilar invagination. The value of the TA of the CVJ is defined by the area determined by three points in the mid-sagittal T2 weighted image: The lowest point of the clivus, the posterior-inferior point of the C2 vertebral body, and the most dorsal indentation point in the ventral aspect of the brain stem (Figure 1). The area can be calculated by most of the viewer applications currently available. The paper by Chang et al. in 2016 analyzed a cohort of BI patients who required odontoidectomy; the TA reportedly represented well the degree of ventral compression and was successfully decreased after decompression and subsequent atlantoaxial fixation.

This study is the first to propose an MRI-based measurement, the TA at the CVJ, for investigation of the degree of compression caused by CVJ pathologies, and also used a large series of patients’ MRIs for validation of its efficacy in quantification and prognostic values.

## 2. Material and Methods

### 2.1. Study Design and Patient Inclusion

This is a retrospective comparison study that included consecutive patients who underwent MRI for disorders of the cervical spine or the CVJ. Patients who had the diagnosis of non-neoplastic CVJ pathologies were extracted from the database for analysis, and compared with other patients who had a normal CVJ (no structural CVJ anomalies on the MRI, and had an ADI < 3 mm on the dynamic lateral radiographs). The cohort of normal CVJ, composed of patients without structural disorders of the CVJ evident on the MRIs, was further divided into a control group and a RA group, according to their underlying medical history of RA. The study was approved by the institutional review board and patients’ informed consent was obtained.

All enrolled patients were grouped into three: CVJ pathology, RA with normal CVJ, and normal control. Exclusion criteria were prior surgery of the cervical spine or the CVJ, infection of the cervical spine or the CVJ, and primary or secondary malignancy of the cervical spine or the CVJ. The TA and other quantitative radiographic measurements were performed on the SmartIris Imaging System (Taiwan Electronic Data Processing Co. Taipei, Taiwan), and interpreted independently by radiologists and neurosurgeons, who were blinded to the patient information. Data were collected and compared between the groups. Some patients of the CVJ pathology group received surgery, including decompression and atlantoaxial fixation [7,8,9,10,11,12], and follow-up MRI was arranged at 1-year and 2-years post-operation. The demographic, clinical, and peri-operative data were also collected. Significant clinical compression at the brain stem or spinal cord included clinical or radiographical myelopathic patients, with either a medical record of neurological deficits caused by myelopathy (clinical myelopathy) or intramedullary increased signal intensity (IISI) on T2-weighted MRIs (radiographical myelopathy).

### 2.2. Radiographical Definition of the Triangular Area of the Cranio-Vertebral Junction (the TA of the CVJ)

In the mid-sagittal T2-weighted MRIs of each patient, a triangular area ventral to the brainstem was delineated on the picture archiving and communication system (PACS) and calculated by its viewer. The TA was defined by three points: The lowest point of the clivus, the posterior-inferior point of the C-2 vertebral body, and the most dorsal indentation point in the ventral aspect of the brain stem [6]. For the surgical cases, adequate decompression and successful reduction was determined by post-operative CT scans and dynamic lateral radiographs. Patients whose post-operative ADI < 3 mm in AAS cases or a realigned posterior cortex of the C2 vertebra in odontoid fracture cases were considered as having complete reduction.

### 2.3. Statistics

Medcalc (Ostend, Belgium) was used for statistical analysis. Descriptive statics were reported as means and standard deviations, and as frequencies and percentages where appropriate. Continuous variables were compared using an unpaired Student *t*-test, and categorical variables were compared using Pearson’s chi-square test. One-way analysis of variance (ANOVA) was used for comparison between multiple continuous variables. Probability values were 2-tailed and an alpha of 0.05 was considered statistically significant. A receiver operating characteristic (ROC) curve analysis was carried out on the TA results. The two factors, the area under the curve (AUC) and cutoff, were used to distinguish the prognostic value in patients with myelopathy. The Youden index ((sensitivity + specificity) − 1) was applied to achieve the best cutoff value.

## 3. Results

### 3.1. Demographics and Normal Values of the Triangular Area

A total of 702 patients were analyzed, including the groups with CVJ pathology (*n* = 129), RA with normal CVJ (*n* = 279), and control (*n* = 294). The mean ages of the three groups were 60.9 ± 16.7, 60.9 ± 12.6, and 57.4 ± 16.2 years, respectively (*p* = 0.009). The RA with normal CVJ group had the fewest male patients than the CVJ pathology group and the control group (15.1%, 34.1%, and 60.9%, respectively, *p* < 0.0001).

The TA of the CVJ was largest in the group with CVJ pathology. The average TA of the CVJ pathology group was significantly larger than the other two groups (1.58 ± 0.47 vs. 1.05 ± 0.26 and 0.96 ± 0.31 cm^2^, *p* < 0.001). Among the patients with CVJ pathology, there were atlantoaxial subluxation in 90 patients, basilar invagination in 3 patients, odontoid fracture in 31 patients, and os odontoideum in 5 patients. The TA of the four subgroups were 1.54 ± 0.42, 1.84 ± 0.27, 1.62 ± 0.56, and 2.15 ± 0.62 cm^2^, respectively, and all significantly larger than the patients with normal CVJ, including the RA with normal CVJ and the control groups (all *p* < 0.0001) (Table 1). Patients with congenital anomalies, including os odontoideum and basilar invagination, had the larger average TA values than degeneration or trauma.

### 3.2. Regression of the Triangular Area after Atlantoaxial Fixation

The surgical success could be validated by the markedly decreased values of the TA of the CVJ. In the study, a total of 81 patients of the CVJ pathology group who received surgery had an averaged TA of 1.67 ± 0.51 cm^2^ pre-operation. The values of the TA after surgery were successfully lowered to 1.34 ± 0.47 cm^2^ and 1.21 ± 0.37 cm^2^ at one-year and two-years post-operation (Table 2). The incremental decrease of the TA values during the follow-up interval was compatible with the common expectation for the continuous shrinkage of the retro-dental soft tissue mass after stabilization of the C1-2 joints. Both the one- and two-year post-operative TAs were significantly smaller than the pre-operative TAs. However, the post-operative two-year TA, 1.21 ± 0.37 cm^2^, was still significantly larger than the RA with normal CVJ and control groups (both *p* < 0.05, not shown in table).

Although reduction of the atlanto-axial subluxation and solid arthrodesis are always the goal of surgery, complete reduction was not always achievable in the series. The post-hoc analysis further divided the surgical cohort into two groups: Partial reduction and complete reduction patients. Patients’ TA of the complete reduction group was significantly lowered from 1.52 ± 0.44 pre-operation to 1.03 ± 0.39 cm^2^ at two-years post-operation, which was similar to that of the control and RA with normal CVJ groups. On the other hand, even though the TA of patients in the partial reduction group demonstrated a significant decrease two-years post-operation (from 1.81 ± 0.52 to 1.41 ± 0.2 cm^2^), the two-year post-operative TA of the partial reduction group was still significantly larger than that of the controls (*p* < 0.05).

### 3.3. The ROC Curve Analysis of the Triangular Area for Myelopathy

**Figure 2:**
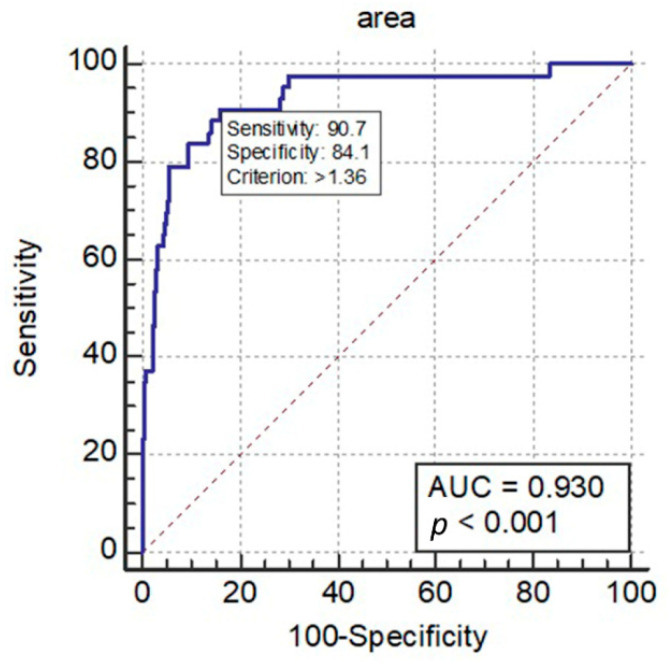
The ROC analysis utilizing the TA for myelopathy detection. As a cut-off value of the TA 1.36 had been identified, and the sensitivity and specificity were 90.7% and 84.1% for myelopathy detection. The AUC was 0.93 (*p* < 0.001).

The ROC curve analysis calculates the agreeable cut-off point of TA-CVJ as 1.36 cm^2^. The Youden index, sensitivity and specificity were 0.75, 0.91, and 0.84, respectively. The AUC was estimated as 0.93 (95% CI: 0.91–0.95; *p* < 0.0001) (Figure 2).

### 3.4. Summary

The TAs of subjects with a normal CVJ were significantly smaller than those with CVJ pathology (1.05 ± 0.26 vs. 1.58 ± 0.47 cm^2^). The TAs decreased after surgical treatment of the CVJ pathology and the extent of reduction could be reflected by the TA decrease. The cut-off value of the TA at 1.36 cm^2^ could be recognized as an indicator of myelopathy from a variety of CVJ pathologies to facilitate surgical treatment decision making.

## 4. Discussion

Among pathologies of the CVJ, RA is frequently associated with retro-odontoid pannus formation that could cause significant neural compression [13,14,15]. Interestingly, the TAs of subjects with RA and a normal CVJ were slightly smaller than those without RA (healthy controls) (0.96 ± 0.31 versus 1.05 ± 0.26 cm^2^). The results implied that the modern medical management of RA, including the use of disease modifying anti-inflammatory drugs (DMARDS), could actually mitigate subsequent development of CVJ disorders [16,17]. At least in RA patients, who were well-controlled and had no pathologies of the CVJ, the mean TA of the CVJ was even less than non-RA patients in the normal control cohort.

**Figure 3:**
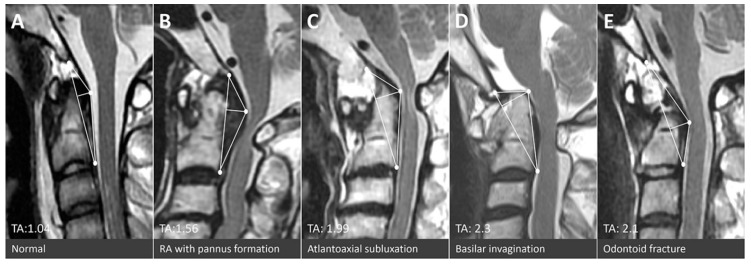
Mid-sagittal T2 weighted magnetic resonance images of patients with (**A**) a normal CVJ, (**B**) rheumatoid arthritis with pannus formation, (**C**) atlantoaxial subluxation, (**D**) basilar invagination, and (**E**) odontoid fracture.

**Figure 4:**
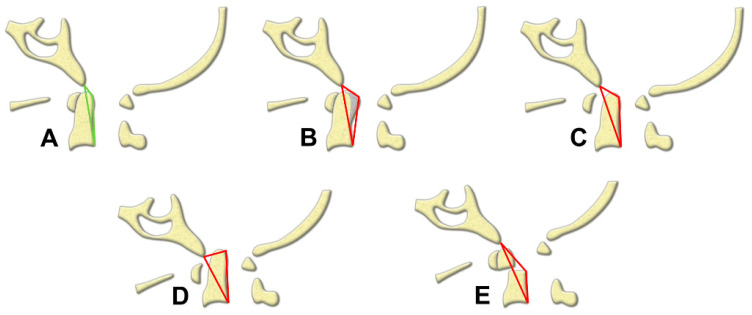
Illustration demonstrating the TA in (**A**) a normal CVJ, (**B**) retro-odontoid soft tissue formation, (**C**) atlantoaxial subluxation, (**D**) basilar invagination, and (**E**) odontoid fracture. Green: normal; Red: abnormal.

The authors advocate use of the TA of the CVJ as an intuitive and direct tool for quantification of compressive lesions on MRIs (Figure 3). The value of the TA is substantially based on two measurements: The base and the height of the designated triangle. The base, the longitudinal axis in normal CVJ anatomy, is mainly determined by the relative position between the skull-C1 complex and the C2 vertebrae. Thus, in circumstances of AA subluxation or odontoid fracture, the length of the base frequently increased and the TA would enlarge simultaneously (Figure 4C,E). The height is determined by the most dorsal indentation point in the ventral aspect of the medulla. Subsequently, the height is influenced by the thickness of the retro-odontoid soft tissues (Figure 4B) or the changes of cranio-medullary angle (Figure 4C,D). The ventral compression to the brain stem could be constantly represented by the simulated triangle in various kinds of CVJ pathologies. Animations of each type of CVJ pathologies and subsequently enlarged TAs are demonstrated in Figure 4. The animated categories of pathologies and deformities of the CVJ also could provide guidance to surgical strategies, which commonly combine decompression, reduction, and fixation.

**Figure 5:**
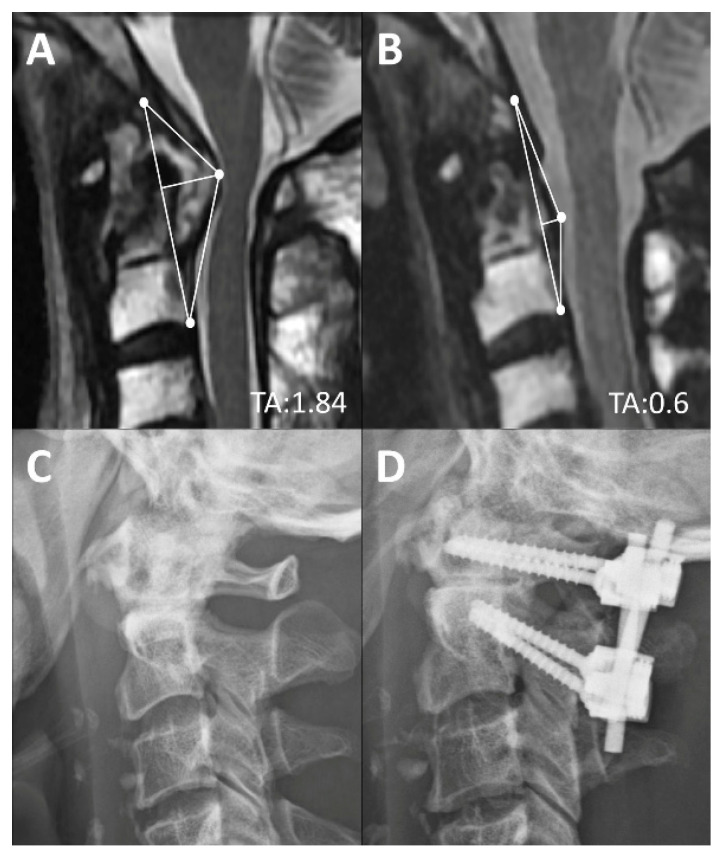
Pre-op (**A**) and 2-year post-op (**B**) mid-sagittal T2 magnetic resonance image of a rheumatoid arthritis patient with retro-odontoid pannus formation. Post-operatively, the base of the TA remained the same and the height decreased from 0.93 to 0.3 cm after regression of the pannus. Pre-op (**C**) and two-year post-op (**D**) lateral radiographs.

The reduction of the TA of the CVJ also could correlate with the success of surgery and relief of myelopathic symptoms. Although in the present study, the two-year post-operative TA remained significantly larger than the normal control or the RA patients with a normal CVJ, significant neurological improvement was observed unanimously. In a subgroup analysis of two-year post-operation data, the key factor of TA normalization was dependent on the completeness of reduction. In patients who had complete reduction of malalignment, the average TA decreased from 1.52 ± 0.44 to 1.03 ± 0.39 cm^2^, which was close to the TA of the control group. In patients who achieved only partial reduction, despite clinical improvement, the TA values decreased from 1.81 ± 0.52 to merely 1.41 ± 0.2 cm^2^ at two-years post-operation. The results were compatible with the concept reportedly emphasized on the importance of reduction and posterior fixation in management of CVJ deformity [18,19,20,21]. In summary, to achieve best decompression, both the base and height of the TA should be addressed by surgery. For example, in complete reduction, the base of the triangle must decrease and the cranio-medullary angle improve immediately. After arthrodesis, the retro-odontoid soft tissue or pannus in patients with RA, continuously regressed at one-year and two-years post-operation, and this was compatible with published literature [22,23,24] (Figure 5).

Commonly used criteria in practice had substantial limitation in decision making. For example, in patients who had RA with AAS, the ADI (less than 3 mm in adults) was a widely accepted screening and diagnostic criterion [4,5]; however, it has quite a few limitations. ADI is uniquely based on the lateral fluoroscopy, which could hardly detect any retro-odontoid soft tissue formation. Furthermore, ADI is not ideal in assessing neurologic outcomes because of its indirect nature that provides no quantification of the nerve compression. Although Boden et al. [4] had proposed ADI >8 mm as a cut-off value for surgical intervention, the sensitivity and specificity for predicting paralysis were only 59% and 58%, while the positive and negative predicting rates were as low as 61% and 56%. Since the incorporation of MRI into pre-operative evaluations in contemporary neurosurgical practice, most of the treatment planning, including the necessity and extent of surgery, are highly dependent on the detailed evaluations made by MRI. In this study, the authors used the ROC curve to analyze the correlation of the TA and myelopathy and yielded a cut-off value of the TA at 1.36 cm^2^, which yielded sensitivity and specificity of 90.7% and 84.1%, respectively. The advances in surgical instrumentations, techniques, and anatomical details provided better outcomes for the patients with CVJ pathologies [25,26]. This TA cut-off value of 1.36 cm^2^ for myelopathy further advanced the management of these patients to provide a critical parameter for surgical timing.

There were limitations to the study. This was a single institute, retrospective, non-randomized, observational study. The control cohort was chosen from patients who underwent MRI of the cervical spine but demonstrated little abnormality. These patients might not be completely healthy controls as they could have been slightly symptomatic or had various other disorders. In addition, the sex distribution was variable within and among the three groups. Although some of the female predominance could be attributed to RA, the value of the TA is also slightly different between sexes in normal subjects. Furthermore, there was heterogeneity in the CVJ pathology cohort. Since the case numbers of AAS and odontoid fracture were substantially larger than that of the BI and the os odontoideum subgroups, there could be selection bias. However, the study tried to utilize the TA to represent the degree of ventral compression to the brain stem before surgery and its regression after surgery. Future investigations should aim to compare the TA between different pathologies when cohort homogeneity could be improved and surgical techniques could be refined.

## 5. Conclusions

Using sagittal MRI, the TA of the CVJ was valued at 1.05 cm^2^ in normal patients, whereas 1.36 cm^2^ could be recognized as a cutoff-point for myelopathy and it is of clinical significance in the decision making and timing for surgical intervention. For patients with CVJ pathologies who underwent atlantoaxial fixation surgery, the TA (1.67 cm^2^) could be reduced by partial and complete reduction (1.41 and 1.03 cm^2^, respectively) post-operation. Therefore, the TA is a valid measurement to quantify compression at the CVJ and to evaluate the efficacy of surgery.

## Figures and Tables

**Table 1:** Comparison of demographics and triangular area.

	Control (Normal CVJ)	RA with Normal CVJ	CVJ Pathology				*p*
*n*	294	279	129				
age	57.4 ± 16.2	60.9 ± 12.6	60.9 ± 16.7				0.009
Sex: Male (%)	179 (60.9%)	42 (15.1%)	44 (34.1%)				<0.0001
Triangular area (cm^2^)	1.05 ± 0.26	0.96 ± 0.31	1.58 ± 0.47				<0.001
pathology			Atlantoaxial subluxation	Basilar invagination	Odontoid fracture	Os odontoideum	
*n*			90	3	31	5	
Triangular area (cm^2^)			1.54 ± 0.42 ^†,‡^	1.84 ± 027 ^†,‡^	1.62 ± 0.56 ^†,‡^	2.15 ± 0.62 ^†,‡^	

^†^*p* < 0.0001 compared to RA with normal CVJ group; ^‡^ *p* < 0.0001 compared to control group; RA: Rheumatoid arthritis; CVJ: Cranio-Vertebral junction.

**Table 2:** Regression of triangular area after surgical fixation of CVJ.

	Triangular Area (cm^2^)		
	Pre-Op	1st Post-Op (Avg 14.6 Months)	2nd Post-Op (Avg 25.8 Months)
Surgery (*n* = 81)	1.67 ± 0.51	1.34 ± 0.47 ^†^	1.21 ± 0.37 ^†^
Complete Reduction (*n* = 47)	1.52 ± 0.44	1.12 ± 0.39 ^†^	1.03 ± 0.39 ^†^
Partial Reduction (*n* = 34)	1.81 ± 0.52	1.59 ± 0.46 ^†^	1.41 ± 0.2 ^†^

^†^*p* < 0.05 compared to pre-op.

## Data Availability

Data sharing is not applicable to this article due to hospital policy.
